# Comparison of navigation systems for total knee arthroplasty: A systematic review and meta-analysis

**DOI:** 10.3389/fsurg.2023.1112147

**Published:** 2023-01-17

**Authors:** Yichao Luan, Huizhi Wang, Min Zhang, Junwei Li, Ningze Zhang, Bolun Liu, Jian Su, Chaohua Fang, Cheng-Kung Cheng

**Affiliations:** ^1^ Key Laboratory of Biomechanics and Mechanobiology, Ministry of Education, Beijing Advanced Innovation Center for Biomedical Engineering, School of Biological Science and Medical Engineering, Beihang University, Beijing, China; ^2^ Engineering Research Center of Digital Medicine, Ministry of Education; School of Biomedical Engineering, Shanghai Jiao Tong University, Shanghai, China; ^3^ Department of Joint Surgery, Ningbo No.6 Hospital, Ningbo, China

**Keywords:** accelerometer-based navigation, component alignment, clinical outcome, surgical duration, meta-analysis, total knee arthroplasty

## Abstract

**Background:**

Component alignment is a crucial factor affecting the clinical outcome of total knee arthroplasty (TKA). Accelerometer-based navigation (ABN) systems were developed to improve the accuracy of alignment during surgery. This study aimed to compare differences in component alignment, clinical outcomes, and surgical duration when using conventional instrumentation (CONI), ABN, and computer navigation (CN) systems.

**Methods:**

A comprehensive literature search was carried out using the Web of Science, Embase, PubMed, and Cochrane databases. Articles that met the eligibility criteria were included in the study. Meta-analyses were performed using the Cochrane Collaboration Review Manager based on Cochrane Review Method. The variables used for the analyses were postoperative clinical outcome (PCO), surgical duration, and component alignment, including the hip-knee-ankle (HKA) angle, coronal femoral angle (CFA), coronal tibial angle (CTA), sagittal femoral angle (SFA), sagittal tibial angle (STA), and the outliers for the mentioned angles. The mean difference (MD) was calculated to determine the difference between the surgical techniques for continuous variables and the odds ratio (OR) was used for the dichotomous outcomes.

**Results:**

The meta-analysis of the CONI and ABN system included 18 studies involving 2,070 TKA procedures, while the comparison of the ABN and CN systems included 5 studies involving 478 TKA procedures. The results showed that the ABN system provided more accurate component alignment for HKA, CFA, CTA, and SFA and produced fewer outliers for HKA, CFA, CTA, and STA. However, while the ABN system also required a significantly longer surgical time than the CONI approach, there was no statistical difference in PCO for the two systems. For the ABN and CN systems, there was no statistical difference in all variables except for the ABN system having a significantly shorter surgical duration.

**Conclusion:**

There was no significant difference in the accuracy of component alignment between the ABN and CN systems, but the ABN approach had a shorter surgical duration and at lower cost. The ABN system also significantly improved the accuracy of component alignment when compared to the CONI approach, although the surgery was longer. However, there was no significant difference in PCO between the CONI, ABN, and CN systems.

## Introduction

Knee osteoarthritis (OA) is one of the most common musculoskeletal disorders, reportedly affecting over 300 million people globally (1). Total knee arthroplasty (TKA) is an effective treatment for severe knee OA and, due to successive developments over the past several decades, this treatment boasts an excellent survival rate (2). However, about 20% of patients report dissatisfaction with postoperative outcomes because of pain and restricted knee function (3). Previous studies demonstrated that the alignment of the knee prosthesis was a key factor influencing postoperative clinical outcomes (4, 5). Malalignment of the prosthesis can affect the mechanics and kinematics of the joint, such as femoral roll-back, tibial rotation, and stress on the ligaments and insert, as well as increasing the wear rate of polyethylene components (6–10). Some prosthetic designs can also take longer to insert, with longer surgical durations being linked to an increased risk of clinical complications and revision (11, 12).

Conventional instrumentation (CONI) is the most widely used apparatus for implanting knee prostheses and uses an intramedullary guide for femoral bone resection and extramedullary tibial bone resection. However, it is difficult to maintain accurate component alignment using this method, with studies showing that about 40% of the coronal and sagittal alignments errors were more than 3 degree which was regarded as the outliers of TKA procedures, and there are more outliers for the femoral component than the tibial component (13). Computer navigation (CN) systems using optical positioning have been developed to improve the accuracy of component alignment during TKA, with results showing fewer outliers and better long-term clinical scores than the CONI system (14, 15). However, computerized systems are relatively novel and complex and so have a higher cost and longer surgical duration (16, 17). Accelerometer-based navigation (ABN) systems are portable surgical navigation systems based on the inertial measurement unit (IMU). ABN has been reported with more accurate alignment than CONI and lower cost than CN systems. Studies have attempted to compare component alignment, surgical duration, and clinical outcomes between the ABN system and CONI, but the results were inconsistent. Li et al. reported that the ABN system could improve the precision of the alignment, but required a longer surgical time (18). Sun et al. suggested that the ABN system could reduce the number of outliers, but there was no significant difference with CONI in terms of the mean values of the alignments, and there was a negligible difference in surgical duration (19). The possible reasons for these discrepancies might be the not latest literature which the studies included in the two analyses were published before 2019. Such previous studies also did not include the CN system in the evaluation, so it is not known how effective this system is by comparison.

Therefore, the purpose of this study is to compare component alignment, surgical duration, and clinical outcomes of TKA procedures performed with conventional instrumentation (CONI), an accelerometer-based navigation (ABN) system, and a computer navigation (CN) system. It was hypothesized that the ABN system would produce a more accurate joint alignment, better clinical outcomes, and require a shorter surgical duration.

## Materials and methods

This systematic review and meta-analysis were based on the Cochrane Review Method and reported using the Preferred Reporting Items for Systematic Reviews and Meta-analyses (PRISMA). The review protocol was registered in the International Prospective register of systematic reviews [CRD42022363153].

### Search strategy and study selection

A comprehensive literature search was carried out using Web of Science, Embase, PubMed, and Cochrane databases. The following terms were used: “arthroplasty, replacement, Knee” and “accelerometer”. For example, the search strategy in PubMed was “[arthroplasty, replacement, knee (MeSH Terms)] AND (accelerometer)”. All publications in English and Chinese up to June 2022 were collected. Relevant studies were identified by the title and abstract of each article. The full text was then reviewed using the eligibility criteria below to confirm whether to include the article in this study.

### Eligibility criteria

Publications were included in this study if they that met the following inclusion criteria: (1) The experimental group used an accelerometer-based navigation (ABN) system in primary TKA. (2) The control group used conventional instrumentation or computer navigation systems in primary TKA. (3) All the TKA procedures were operated by performed using mechanical alignment. (4) At least one of the following outcomes was included: surgical duration, postoperative clinical outcomes (PCO), hip-knee-ankle angle (HKA), coronal femoral angle (CFA), coronal tibial angle (CTA), sagittal femoral angle (SFA), sagittal tibial angle (STA), and the outliers for the mentioned angles, (5) The studies were randomized controlled trials (RCT) or prospective and retrospective nonrandomized controlled trials (nRCT).

### Quality assessment

The risk of bias in the included studies was evaluated by two reviewers according to the Cochrane risk of bias tool. The assessed parameters included randomization procedure, allocation concealment, blinding of patients and surgeons, blinding of outcome assessors, selective outcome reporting, incomplete outcome data, and other biases. Each parameter was judged as a having high, low, or unclear risk of bias by the two reviewers independently. A discussion proceeded in case of any disagreements in the bias judgment.

### Data extraction and analysis

The data was extracted from the included studies by the two reviewers. The information on the publications included the first author, year of publication, sample size, ABN device, and implant system. Patient information included age, gender, and body mass index (BMI). The primary outcomes were postoperative alignment parameters, including HKA, CFA, CTA, SFA, STA, and their outliers. The rotational alignment was not extracted from the studies since the rotational alignment was not considered in any of the currently approved ABN systems. The secondary outcome was the postoperative clinical outcome. The surgical duration was regarded as the tertiary outcome. Any discrepancies in the extracted data were resolved by discussion among the reviewers.

### Statistical analysis

A meta-analysis of the comparison between the ABN and CONI systems and between the ABN and CN systems was performed using the Cochrane Collaboration Review Manager 5.4 software. For continuous variables, such as the alignment angles, clinical outcomes, and surgical duration, the mean difference (MD) was calculated with the Inverse-Variance method to show the difference between the surgical techniques. The odds ratio (OR) was used with the Mantel-Haenszel method to determine discrepancies between the surgical techniques for the dichotomous outcomes, such as the number of outliers of the measured alignment angles. The 95% confidence interval (CI) of the MD and OR was calculated for each study. The *I*^2^ statistic was used to assess the heterogeneity. A fixed model was utilized for the variables where *I*^2^ < 50% and a random model was used where *I*^2^ > 50%. Values of *P* < 0.05 were considered statistically significant.

## Results

### Study selection

**Figure 1:**
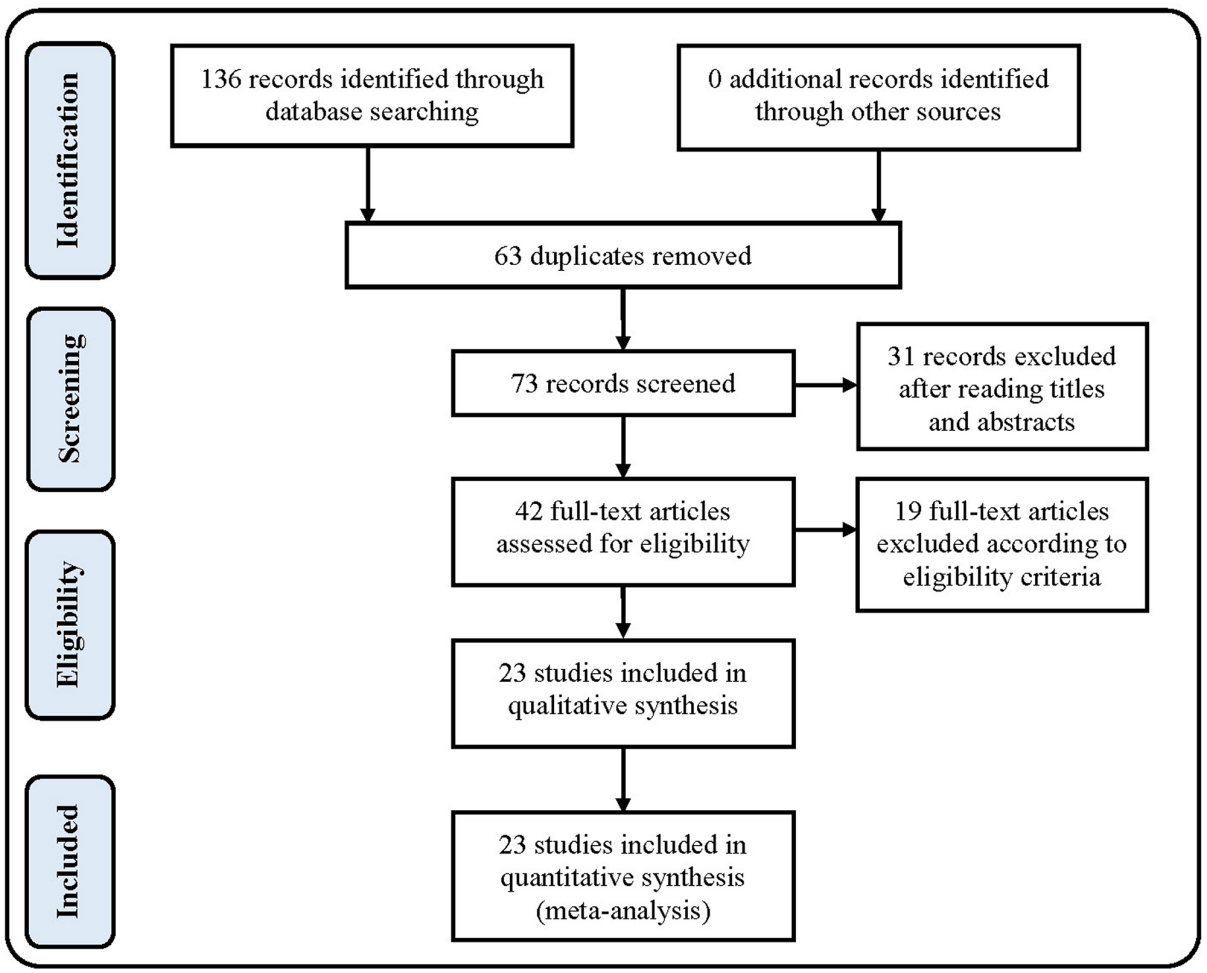
Flow chart of screened publications.

The screening process of the included studies is shown in Figure 1. One hundred and thirty-six publications were identified through the database search, sixty-three of which were excluded because of duplicates and another thirty-one were excluded after reading the title and abstract. Of the remaining forty-two full-text articles, twenty-three articles (20–42) met the eligibility criteria and were included in this study. eighteen articles compared the ABN and CONI systems, and five articles compared the ABN and CN systems.

### Characteristics

Table 1 details the characteristics of the studies for the meta-analysis of the ABN and CONI systems. The eighteen studies included in this report assessed a total of 2,070 TKA procedures, of which 947 used the ABN system and the remaining 1,123 patients underwent conventional surgery. The ABN system used in most cases was either iAssist® (Zimmer Biomet Inc., Warsaw, IN, USA) or KneeAlign® (OrthAlign Inc., Columbia, CA, USA), while one study used i-Join® (i-Join Medical Technology Inc., Shanghai, China). The mean age of the patients in the ABN group and CONI group was 70.58 and 70.24 years old, respectively, and the average BMI of the patients was 27.15 and 27.77, respectively. All of the included studies reported at least one primary outcome, and 7 studies also reported the PCO scores (secondary outcome). 11 studies reported the surgical duration (tertiary outcome).

**Table 1:** Studies included in the meta-analysis of the ABN and CONI systems.

Year	Author	Implant	System	Number		Age (years)		BMI		Follow-up (months)		Outcomes		
				ABN	CONI	ABN	CONI	ABN	CONI	ABN	CONI	Primary	Second	Tertiary
2022	Wang	Vanguard	iAssist	25	24	67.7 ± 7.9	66.7 ± 6.1	25.9 ± 3.4	27.0 ± 3.9	NA	NA	HKA, HKAOutliers, CFA, CFAOutliers, CTA, CTAOutliers	NA	NA
2022	Jagadeesh	NA	KneeAlign	35	35	61.9	63.9	NA	NA	24.0	24.0	HKA, CFA, CTA, STA	KSS, KFS, OKS	SD
2021	Tsuda	Persona	iAssist	42	41	74.2 ± 8.1	75.5 ± 9.0	25.4 ± 4.0	26.2 ± 4.4	6.0	6.0	HKAOutliers, CFAOutliers, CTAOutliers	ROM, KSS, KFS, EQ-5D, OKS	SD
2021	Laoruengthana	NexGen	iAssist	44	57	64.9 ± 6.2	62.8 ± 7.9	26.8 ± 4.3	26.8 ± 3.8	NA	NA	HKA, HKAOutliers, CFA, CFAOutliers, CTA, CTAOutliers, SFA, SFAOutliers, STA, STAOutliers	NA	SD
2021	Gao	NA	iAssist	24	78	71.0 ± 6.8	69.2 ± 7.3	27.8 ± 2.9	27.4 ± 3.5	21.9	21.6	HKA, HKAOutliers, CFA, CFAOutliers, CTA, CTAOutliers, SFA, SFAOutliers, STA, STAOutliers	ROM, KSS, KFS	SD
2020	Minoda	Vanguard	KneeAlign	50	50	76 ± 5	74 ± 7	26.5 ± 4.4	27.4 ± 4.2	6.0	6.0	HKA, HKAOutliers, CFA, CFAOutliers, CTA, CTAOutliers	KSS, KFS, EQ-5D	NA
2020	Lai	NexgenLPS	iAssist	38	44	68.0	69.1	NA	NA	NA	NA	HKA, HKAOutliers, CFA, CFAOutliers, CTA, CTAOutliers, SFA, STA	NA	SD
2019	Zhu	Persona\NexGen	iAssist	28	110	67.0	68.2	33.2	33.8	NA	NA	CFA, CFAOutliers, CTA, CTAOutliers	NA	NA
2019	Xu	GenesisII	i-Join	39	40	65.3 ± 6.8	65.3 ± 7.6	NA	NA	NA	NA	HKA, HKAOutliers, CFA, CFAOutliers	NA	SD
2019	Ueyama	Vanguard	KneeAlign	78	81	77.8 ± 6.4	78.5 ± 4.5	25.4 ± 3.7	24.2 ± 2.8	NA	NA	CFA, CFAOutliers, CTA, CTAOutliers, SFA, SFAOutliers, STA, STAOutliers	NA	SD
2019	Gao	NA	iAssist	41	41	67.9 ± 7.3	67.7 ± 7.1	26.8 ± 2.6	26.6 ± 1.5	21.2	20.9	HKA, HKAOutliers, CFA, CFAOutliers, CTA, CTAOutliers, SFA, SFAOutliers, STA, STAOutliers	KSS, KFS	NA
2018	Matsumoto	Vanguard	KneeAlign	50	50	74.7	73.1	25.4	26.4	NA	NA	HKA, HKAOutliers, CFA, CFAOutliers, CTA, CTAOutliers, SFA, SFAOutliers, STA, STAOutliers	NA	NA
2017	Ueyama	Vanguard	KneeAlign	67	75	76.9 ± 4.8	78.1 ± 5.1	26 ± 3.8	25.1 ± 4.4	10.8	21.4	HKA, CFA, CFAOutliers, CTA, CTAOutliers, SFA, SFAOutliers, STA, STAOutliers	ROM, KSS, KFS	SD
2017	Ikawa	Vanguard	KneeAlign	121	120	74.0 ± 6.8	74.1 ± 6.8	26.1 ± 3.7	26.8 ± 4.1	NA	NA	HKA, CFA, CFAOutliers	NA	SD
2017	Gharaibeh	Legion\Score	KneeAlign	89	90	69.2 ± 8.7	69 ± 8.3	29.2 ± 4.8	29.6 ± 5.4	NA	NA	HKA, HKAOutliers, CFA, CFAOutliers, CTA, CTAOutliers, SFA, SFAOutliers, STA, STAOutliers	NA	NA
2016	Thiengwittayaporn	Nexgen	iAssist	40	40	68.0 ± 8.0	65.9 ± 6.3	26.6 ± 3.7	26.2 ± 3.2	NA	NA	HKA, HKAOutliers, CFA, CFAOutliers, CTA, CTAOutliers, SFA, SFAOutliers, STA, STAOutliers	NA	SD
2016	Liow	NA	iAssist	92	100	65.0 ± 6.2	66.3 ± 7.3	28.1 ± 4.9	27.6 ± 5.3	6.0	6.0	HKA, CFA, CTA	KSS, KFS, OKS	SD
2014	Nam	NA	KneeAlign	47	47	67.1 ± 7.5	66.1 ± 10.1	31.1 ± 5.9	31.2 ± 5.6	NA	NA	HKA, HKAOutliers, CFAOutliers, CTA, CTAOutliers, STA, STAOutliers	NA	NA

HKA, hip-knee ankle angle; CFA, coronal femoral angle; CTA, coronal tibial angle; SFA, sagittal femoral angle; STA, sagittal tibial angle; KSS, knee society score; KFS, knee functional score; OKS, oxford knee score; ROM, range of motion; EQ-5D, EuroQol-5Dimensions; SD, surgical duration.

Table 2 shows the characteristics of the studies for the meta-analysis of the ABN and CN systems. The five studies included in this report assessed a total of 478 TKA procedures, of which 238 used the ABN system and the remaining 240 patients underwent computer navigation surgery. The iAssist® and KneeAlign® systems were used in the ABN group. The computer navigation system included AchieveCAS® (Smith & Nephew Inc., Memphis, Tennessee, USA), Ci Mi TKR® (BrainLab/DePuy Orthopaedics Inc. Munich, Germany), and OrthoPilot® (B. Braun Aesculap Inc., Tuttlingen, Germany). The mean age of the patients in the ABN Group and CN Group was 64.66 and 64.72 years old, respectively, and the patients had an average BMI of 28.72 and 29.86, respectively. All of the included studies reported at least one primary outcome, 3 studies described the PCO scores (secondary outcome), and 3 studies reported the surgical duration (tertiary outcome).

**Table 2:** Studies included in the meta-analysis of the ABN and CN systems.

Year	Author	Implant	ABN System	CN System	Number		Age (years)		BMI		Follow-up (months)		Outcomes		
					ABN	CN	ABN	CN	ABN	CN	ABN	CN	Primary	Second	Tertiary
2022	Swamy	GenesisII/Columbus	KneeAlign	OrthoPilot	50	50	59.4 ± 8.1	60.7 ± 4.9	29.4 ± 5.7	30.0 ± 4.1	12	12	HKA, HKAOutliers, CFA, CFAOutliers, CTA, CTAOutliers, SFA, SFAOutliers, STA, STAOutliers	ROM, KSSs, KSSe, KFS, OKS	NA
2021	Wang	NA	iAssist	OrthoPilot	40	42	66.3 ± 6.1	67.2 ± 7.0	26.6 ± 3.9	27.7 ± 2.8	NA	NA	HKA, CFA, CFAOutliers, CTA, CTAOutliers	NA	SD
2021	Tsubosaka	Persona/e-motion	iAssist	OrthoPilot	30	30	75.8 ± 7.0	74.2 ± 9.4	26.0 ± 3.3	26.4 ± 4.5	12.0	12.0	HKA, CTA, STA	ROM, KSSs, KSSe, KFS	NA
2016	Goh	NA	iAssist	Ci Mi TKR	38	38	63.9 ± 7.4	64.9 ± 7.1	28.9 ± 5.7	28.4 ± 5.1	6.0	6.0	HKA, CFA, CFAOutliers, CTA, CTAOutliers	ROM, KSS, KFS, OKS	SD
2013	Nam	NA	KneeAlign	Achieve CAS	80	80	63.3 ± 9.0	62.3 ± 9.8	30.3 ± 5.8	32.9 ± 7.2	NA	NA	HKA, CFA, CFAOutliers, CTA, CTAOutliers	NA	SD

HKA, hip-knee ankle angle; CFA, coronal femoral angle; CTA, coronal tibial angle; SFA, sagittal femoral angle; STA, sagittal tibial angle; ROM, range of motion; KSSs, knee society score satisfaction; KSSe, knee society score expectation; KFS, knee functional score; OKS, oxford knee score; KSS, knee society score; SD, surgical duration.

### Risk of bias

**Figure 2:**
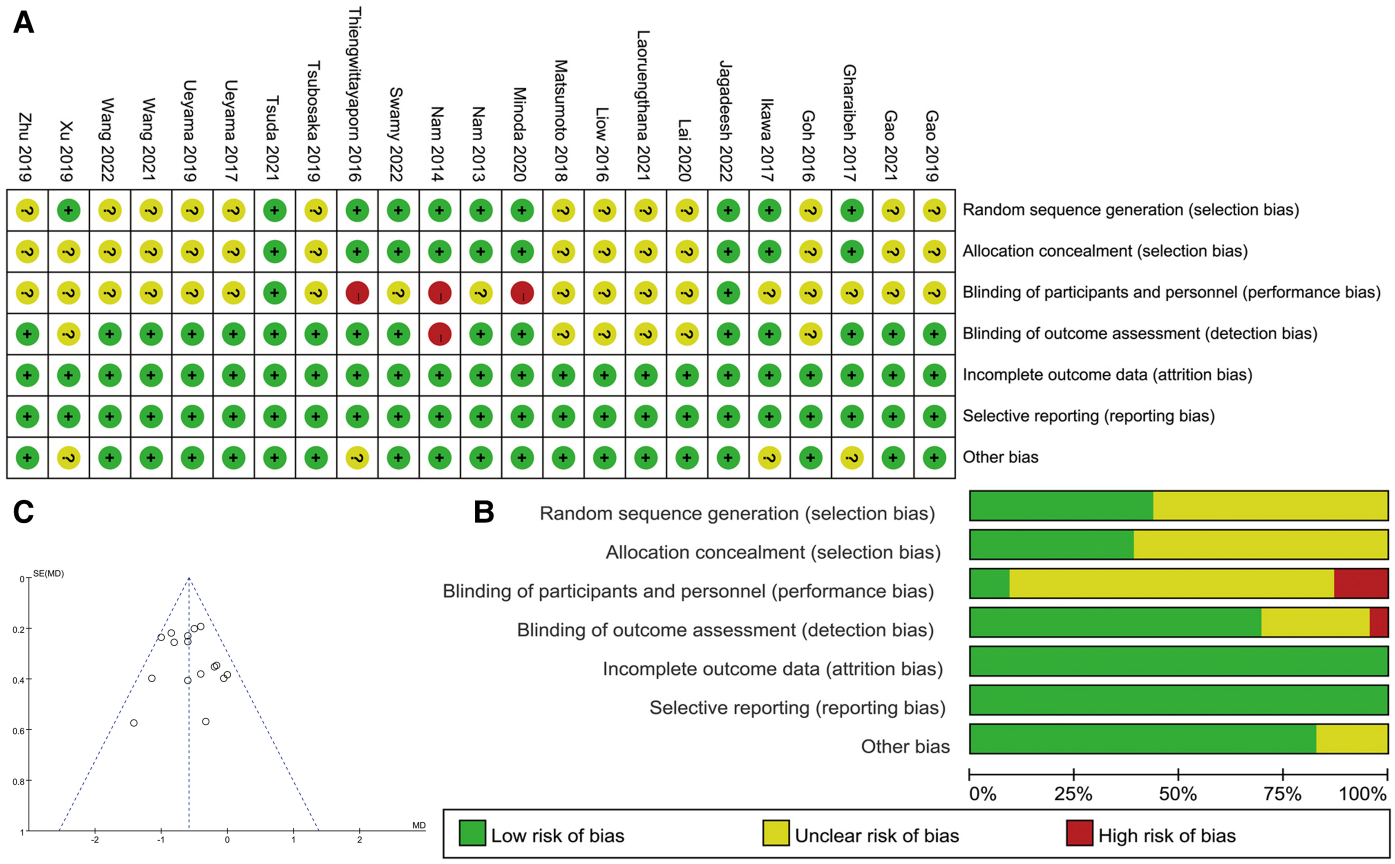
(**A**) Risk of bias for the included studies (+: low risk, ?: unclear risk, −: high risk); (**B**) overall risk of bias for all studies; (**C**) the funnel plots of the coronal femoral angle.

The risk of bias for the included studies is shown in Figure 2A. The risk of selection bias and performance bias was not unclear because information on the randomization process and blinding of participants and personnel in some of the included studies were not described in sufficient detail. All studies reported the complete outcome data. The overall risk of bias for all studies is shown in Figure 2B, with each measure being presented as a percentage. The funnel plots of the coronal femoral angle were shown in Figure 2C which indicated a low publication bias.

### Primary outcome

**Figure 3:**
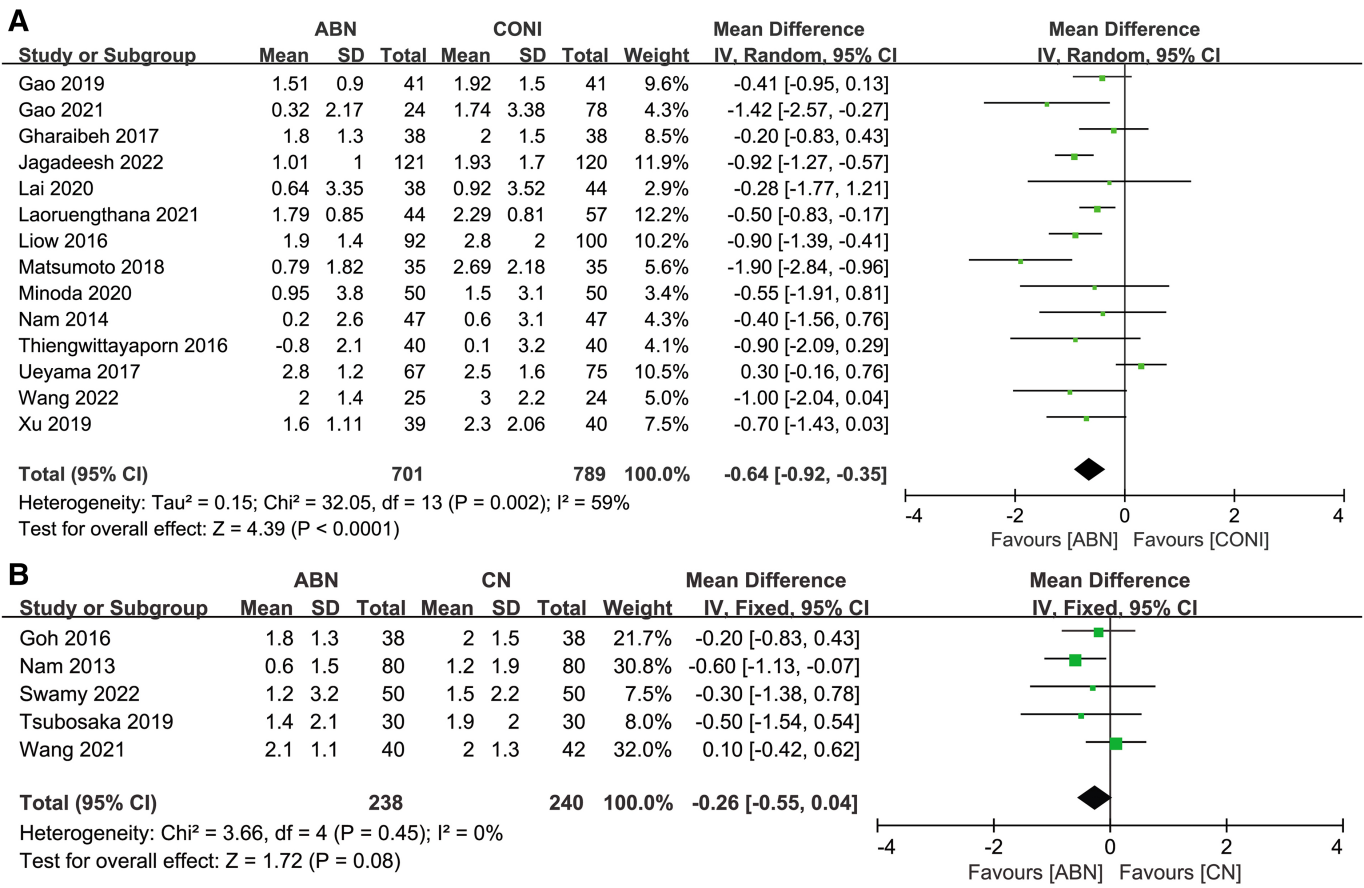
Forest plot of hip-knee-ankle (HKA) angle. (**A**) Accelerometer-based navigation (ABN) system vs. conventional instrumentation (CONI); (**B**) ABN system vs. computer navigation (CN) system.

Fourteen studies reported the postoperative hip-knee-ankle (HKA) angle when using the ABN system or CONI approach. The results suggest that the ABN system allows for more accurate lower limb alignment (Figure 3A, MD: −0.64, 95% CI: −0.92 to −0.35, *P* < 0.0001, *I*^2^ = 59%). The results of five studies comparing the HKA when using the ABN and CN systems did not show any significant difference between the two alignment techniques (Figure 3B, MD: −0.26, 95% CI: −0.55 to −0.04, *P* = 0.08, *I*^2^ = 0%).

**Figure 4:**
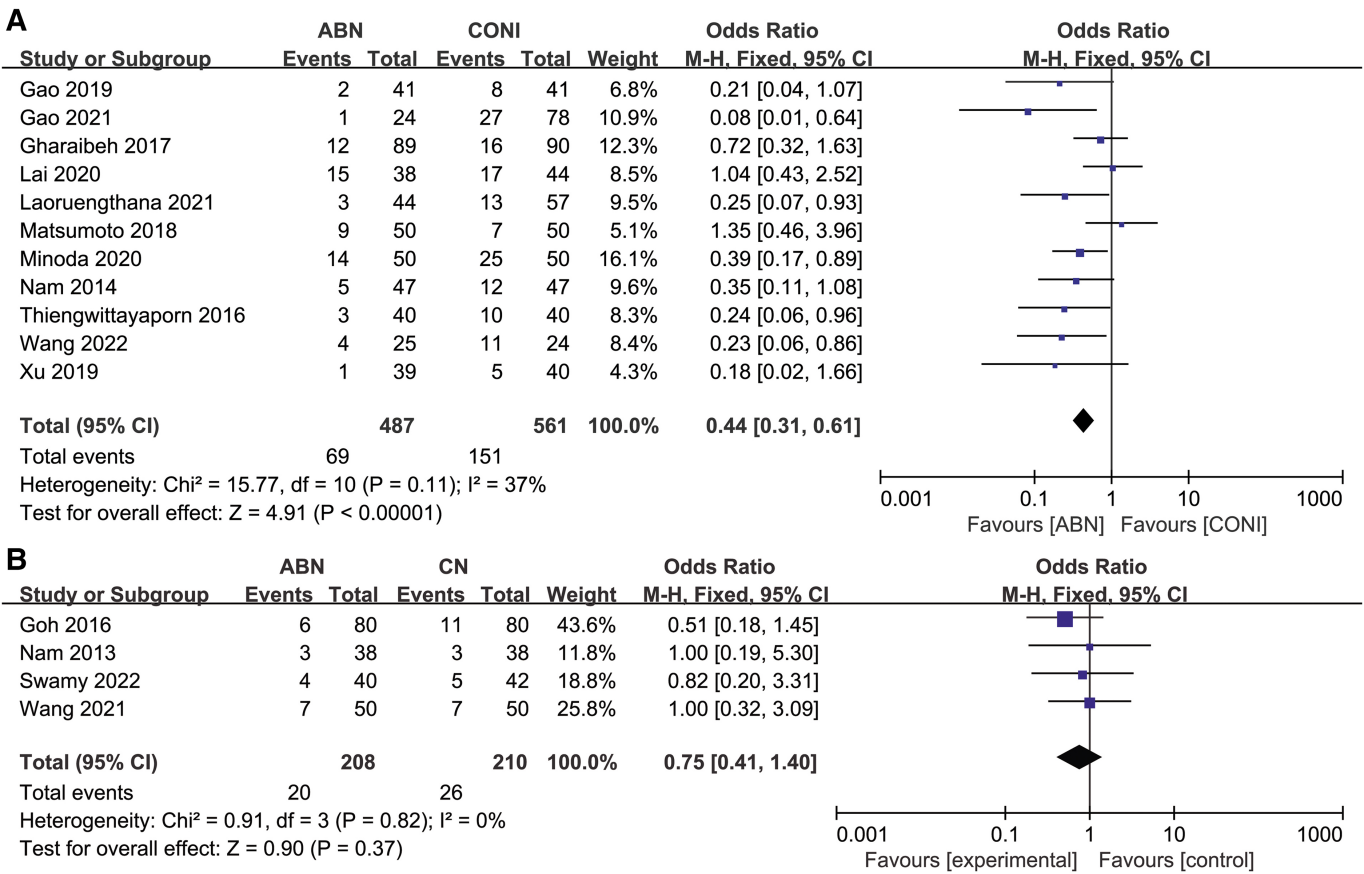
Forest plot of HKA outliers. (**A**) Accelerometer-based navigation (ABN) system vs. conventional instrumentation (CONI); (**B**) ABN system vs. computer navigation (CN) system.

Details of outliers for HKA measurements for the ABN and CONI groups were documented in 11 articles, and the results suggested fewer outliers when using the ABN system (Figure 4A, OR:0.44, 95% CI: 0.31 to 0.61, *P* < 0.00001, *I*^2^ = 37%). Only four studies compared HKA outliers between the ABN and CN systems, and no significant differences were re-ported (Figure 4B, OR:0.75, 95% CI: 0.41 to 1.40, *P* = 0.37, *I*^2^ = 0%).

**Figure 5:**
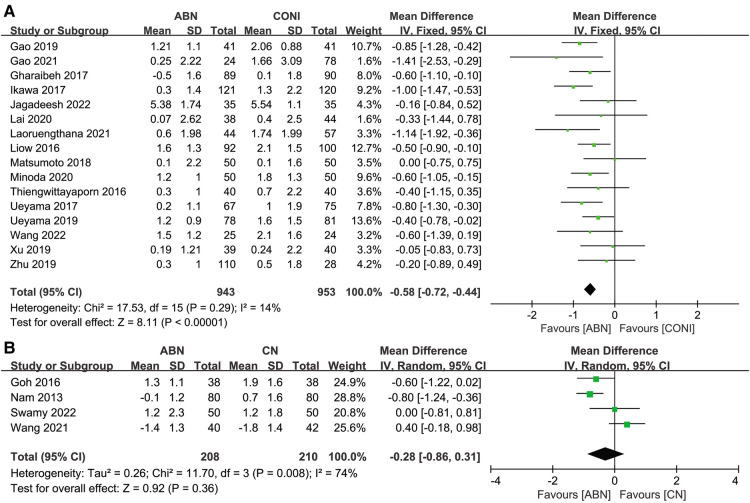
Forest plot of coronal femoral angle (CFA). (**A**) Accelerometer-based navigation (ABN) system vs. conventional instrumentation (CONI); (**B**) ABN system vs. computer navigation (CN) system.

Sixteen studies compared the coronal femoral angle (CFA) when using the ABN and CONI approaches, with the results showing that the ABN system provides more accurate alignment (Figure 5A, MD: −0.58, 95% CI: −0.72 to −0.44, *P* < 0.00001, *I*^2^ = 14%). A further four studies compared the CFA for the ABN and CN systems, and no significant difference was found (Figure 5B, MD: −0.28, 95% CI: −0.86 to 0.31, *P* = 0.36, *I*^2^ = 74%).

**Figure 6:**
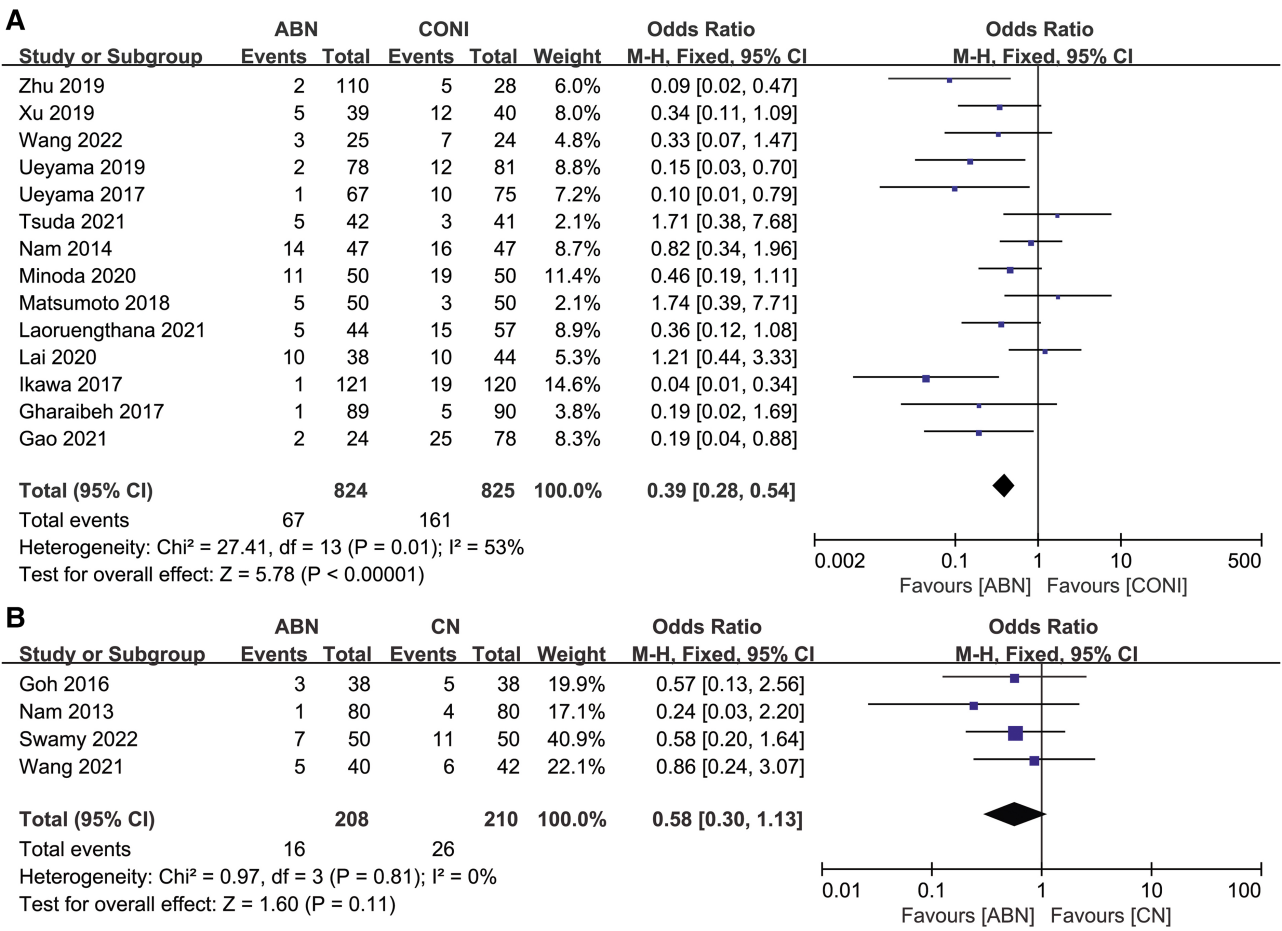
Forest plot of CFA outliers. (**A**) Accelerometer-based navigation (ABN) system vs. conventional instrumentation (CONI); (**B**) ABN system vs. computer navigation (CN) system.

Fourteen studies assessed CFA outliers recorded following surgery using the ABN and CONI approach. The results showed fewer outliers in the ABN group (Figure 6A, OR: 0.39, 95% CI: 0.28 to 0.54, *P* < 0.00001, *I*^2^ = 53%). Only four articles assessed differences in CFA outliers between the ABN and CN groups, with the results showing no significant difference between the two groups (Figure 6B, OR: 0.58, 95% CI: 0.30 to 1.13, *P* = 0.11, *I*^2^ = 0%).

**Figure 7:**
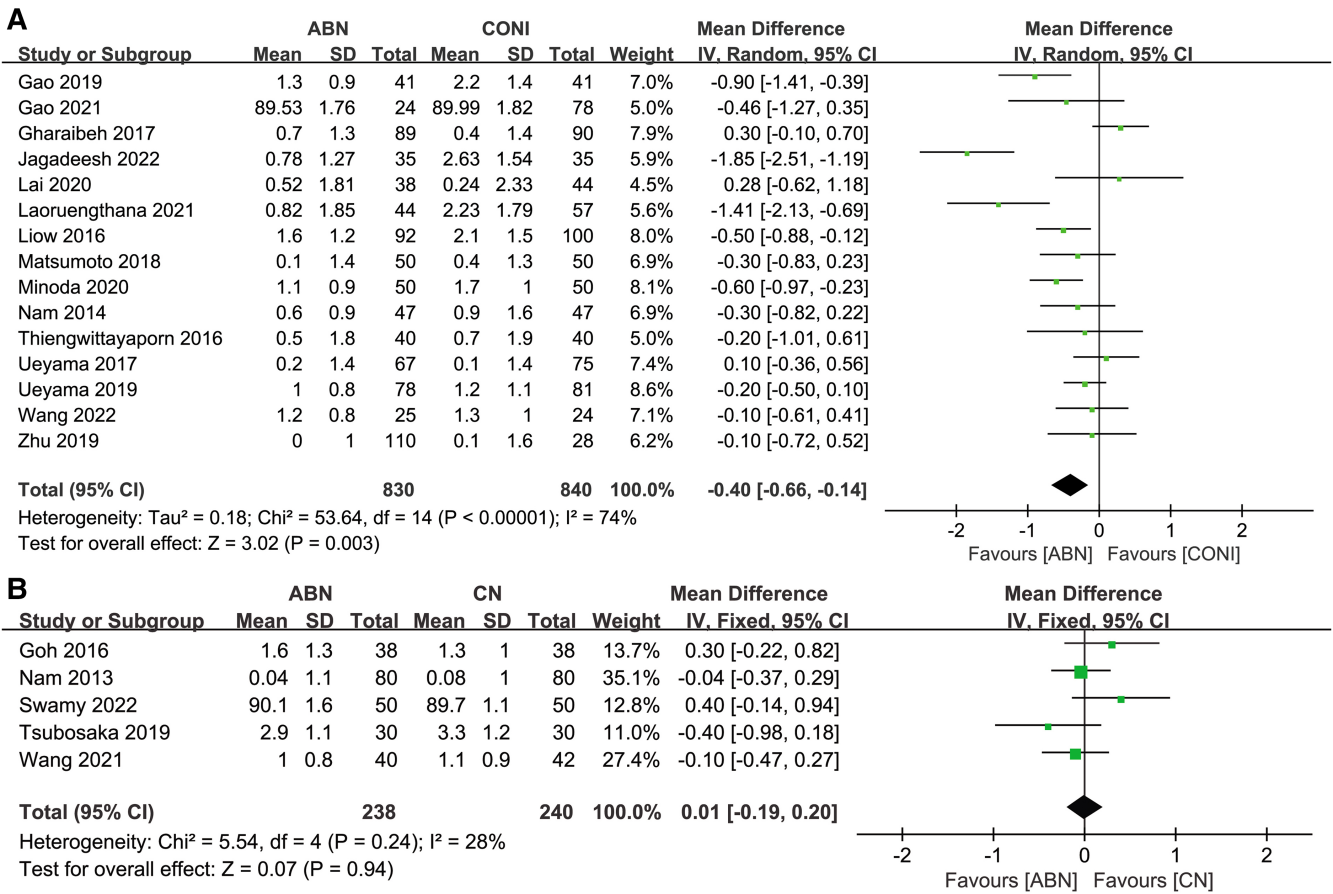
Forest plot of coronal tibial angle (CTA). (**A**) Accelerometer-based navigation (ABN) system vs. conventional instrumentation (CONI); (**B**) ABN system vs. computer navigation (CN) system.

Fifteen studies reported on the measurements for the coronal tibial angle (CTA) following ABN and CONI. As with previous measurements, the ABN system was capable of more accurate alignment (Figure 7A, MD: −0.40, 95% CI: −0.66 to −0.14, *P* = 0.003, *I*^2^ = 74%). Five studies recorded the CTA following ABN and CN, with no significant difference reported be-tween the results for the two systems (Figure 7B, MD: 0.01, 95% CI: −0.19 to 0.20, *P* = 0.94, *I*^2^ = 28%).

**Figure 8:**
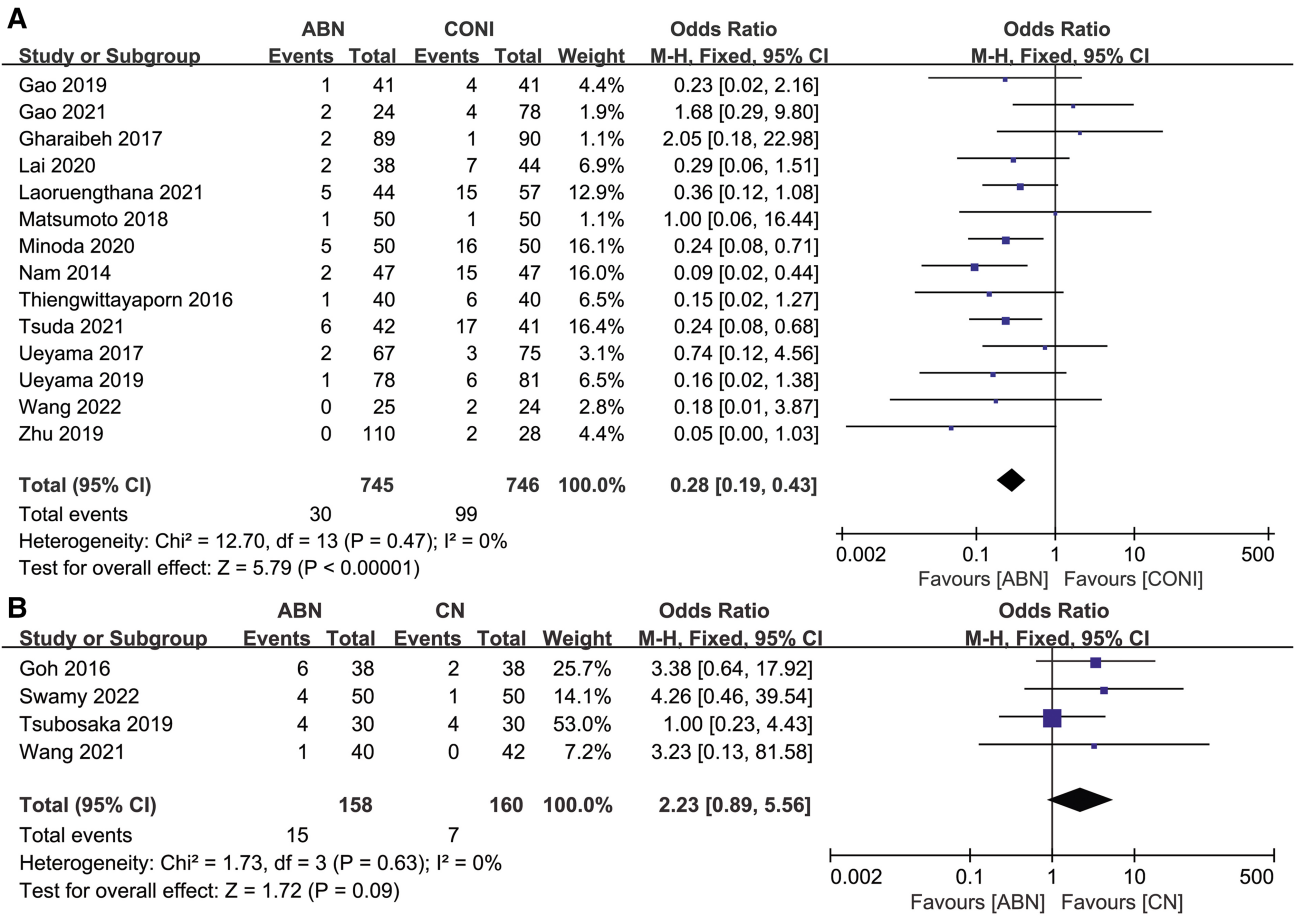
Forest plot of CTA outliers. (**A**) Accelerometer-based navigation (ABN) system vs. conventional instrumentation (CONI); (**B**) ABN system vs. computer navigation (CN) system.

Fourteen articles presented the CTA outliers after performing surgery using ABN and CONI, with the fewer outliers reported for the ABN group (Figure 8A, OR: 0.28, 95% CI: 0.19 to 0.43, *P* < 0.00001, *I*^2^ = 0%). Four studies compared CTA outliers for the ABN and CN groups, with no significant difference reported between the two groups (Figure 8B, OR:2.23, 95% CI: 0.89 to 5.56, *P* = 0.09, *I*^2^ = 0%).

**Figure 9:**
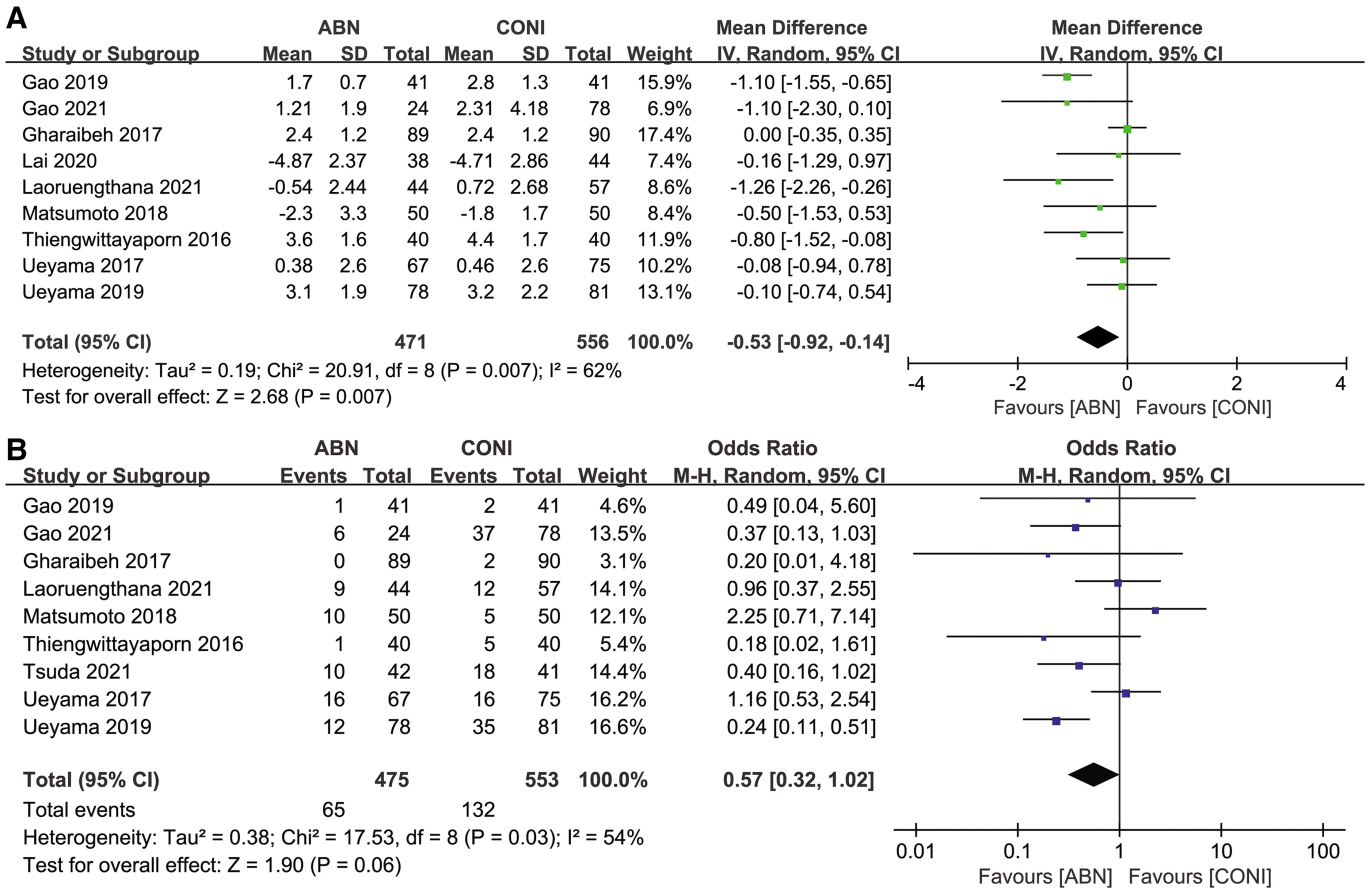
Forest plot of sagittal femoral angle (SFA). (**A**) And SFA outliers (**B**) between accelerometer-based navigation (ABN) system and conventional instrumentation (CONI).

Nine articles reported on the sagittal femoral angle (SFA) after using the ABN and CONI approach. As expected, the ABN system provided more accurate alignment (Figure 9A, MD: −0.53, 95% CI: −0.92 to −0. 14, *P* = 0.007, *I*^2^ = 62%). However, there was no significant difference in SFA outliers for the two approaches (Figure 9B, OR:0.57, 95% CI: 0.32 to 1.02, *P* = 0.06, *I*^2^ = 54%). Only one study compared the SFA and outliers for the ABN and CN systems, with the results showing no significant difference between the two groups (*P* = 0.51) (33).

**Figure 10:**
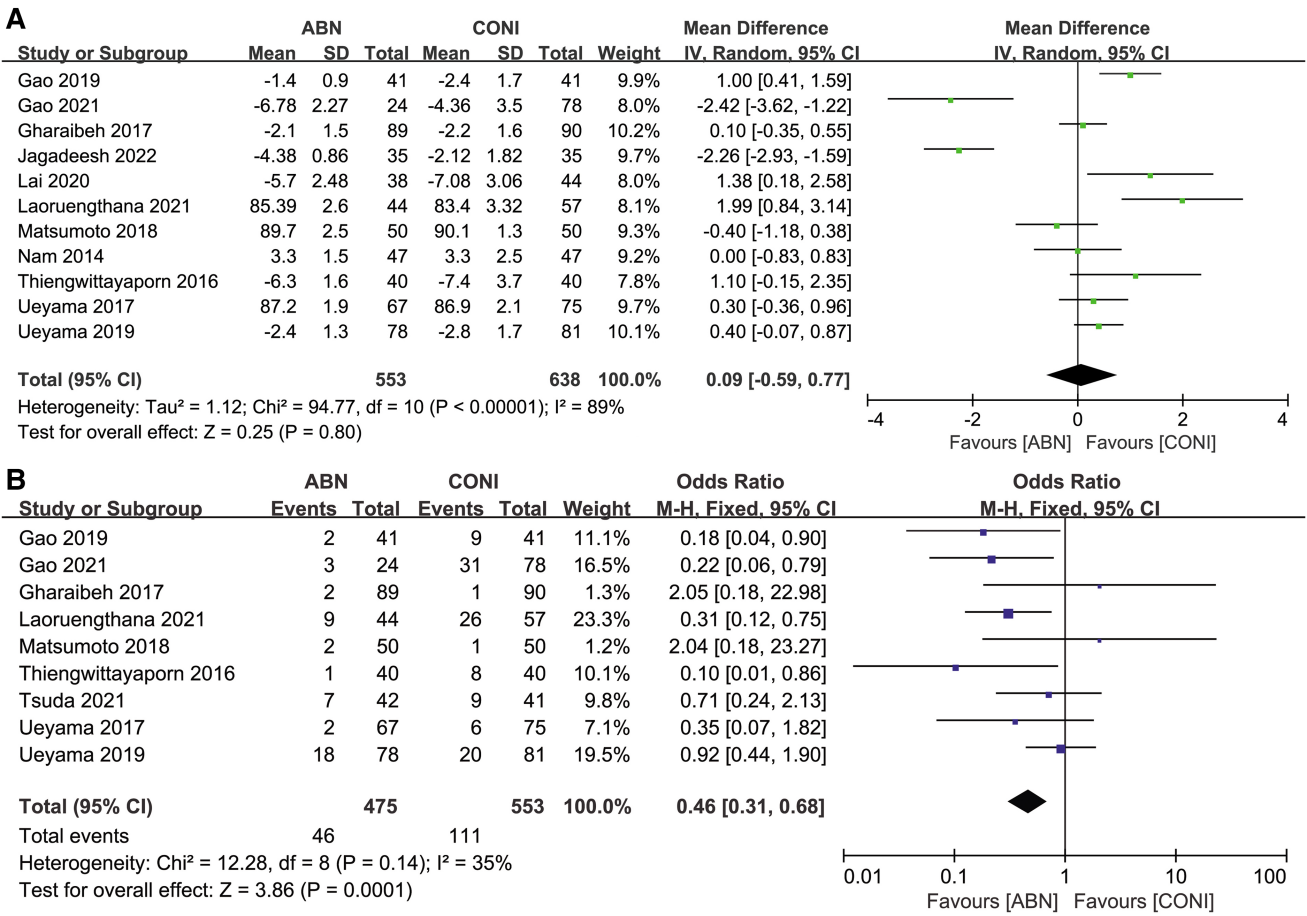
Forest plot of sagittal tibial angle (STA). (**A**) And STA outliers (**B**) between accelerometer-based navigation (ABN) system and conventional instrumentation (CONI).

Eleven articles assessed the sagittal tibial angle (STA) recorded wen using the ABN and CONI approach. The differences between the two groups were not significant (Figure 10A, MD: 0.09, 95% CI: −0.59 to 0.77, *P* = 0.80, *I*^2^ = 89%). Nine studies reported on STA outliers and the results suggested fewer outliers with the ABN approach (Figure 10B, OR:0.46, 95% CI: 0.31 to 0.68, *P* = 0.0001, *I*^2^ = 35%). Only 1 study reported on the STA and its outliers following the ABN and CN approaches. No significant difference was identified for the STA (*P* = 0.36) or its outliers (*P* = 0.15) (33).

### Secondary outcome

**Figure 11:**
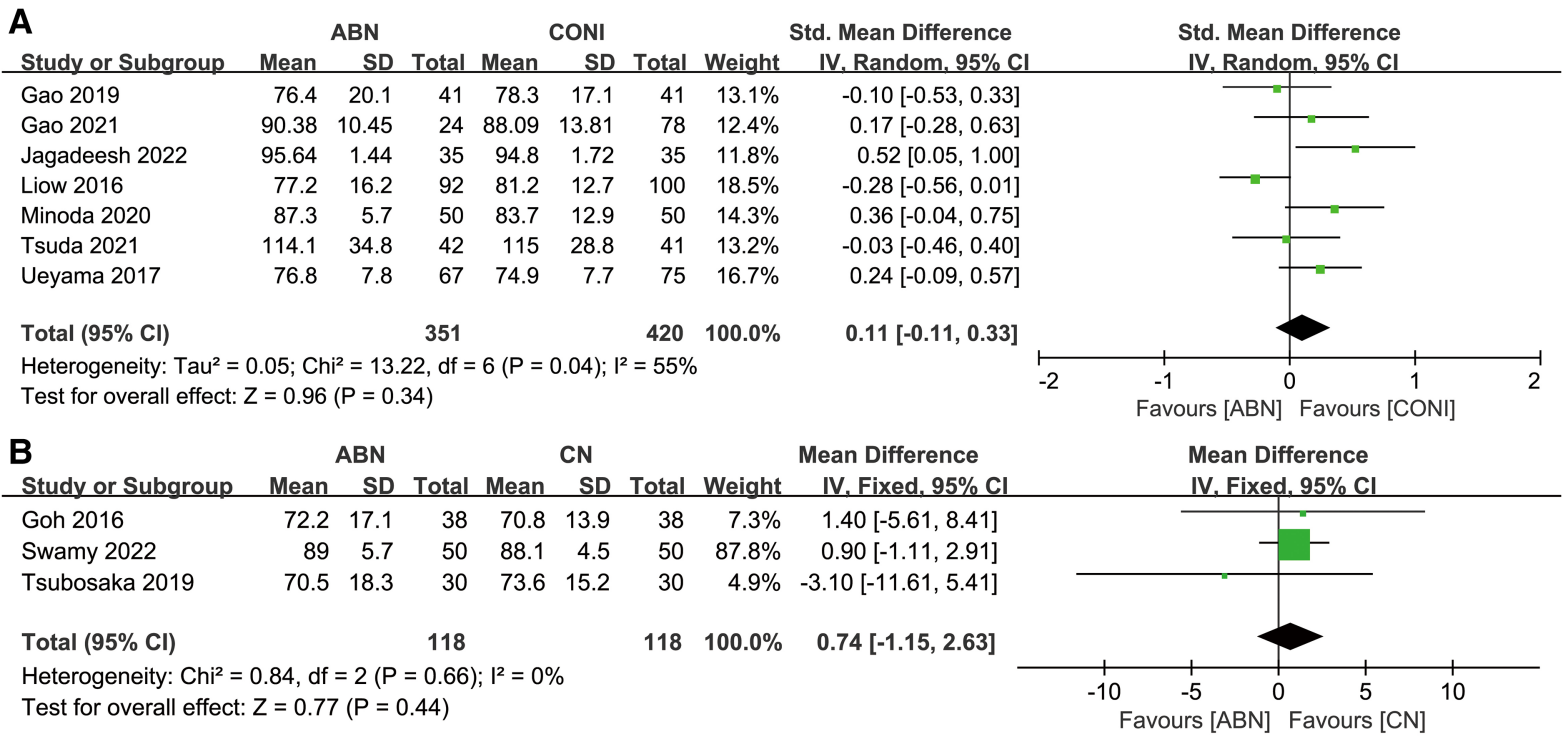
Forest plot of postoperative clinical outcomes (PCO). (**A**) Accelerometer-based navigation (ABN) system vs. conventional instrumentation (CONI); (**B**) ABN system vs. computer navigation (CN) system.

For the secondary outcome in this current study, seven articles report on short-term postoperative clinical outcomes (PCO) following surgery using the ABN and CONI approaches. Analysis of the results did not identify any significant difference between the two groups (Figure 11A, MD:0.11, 95% CI: −0.11 to 0.33, *P* = 0.34, *I*^2^ = 55%). Three articles compared the short-term PCO for the ABN and CN groups. The difference between the groups was not significant (Figure 11B, MD: 0.74, 95% CI: −1.15 to 2.63, *P* = 0.44, *I*^2^ = 0%).

### Tertiary outcome

**Figure 12:**
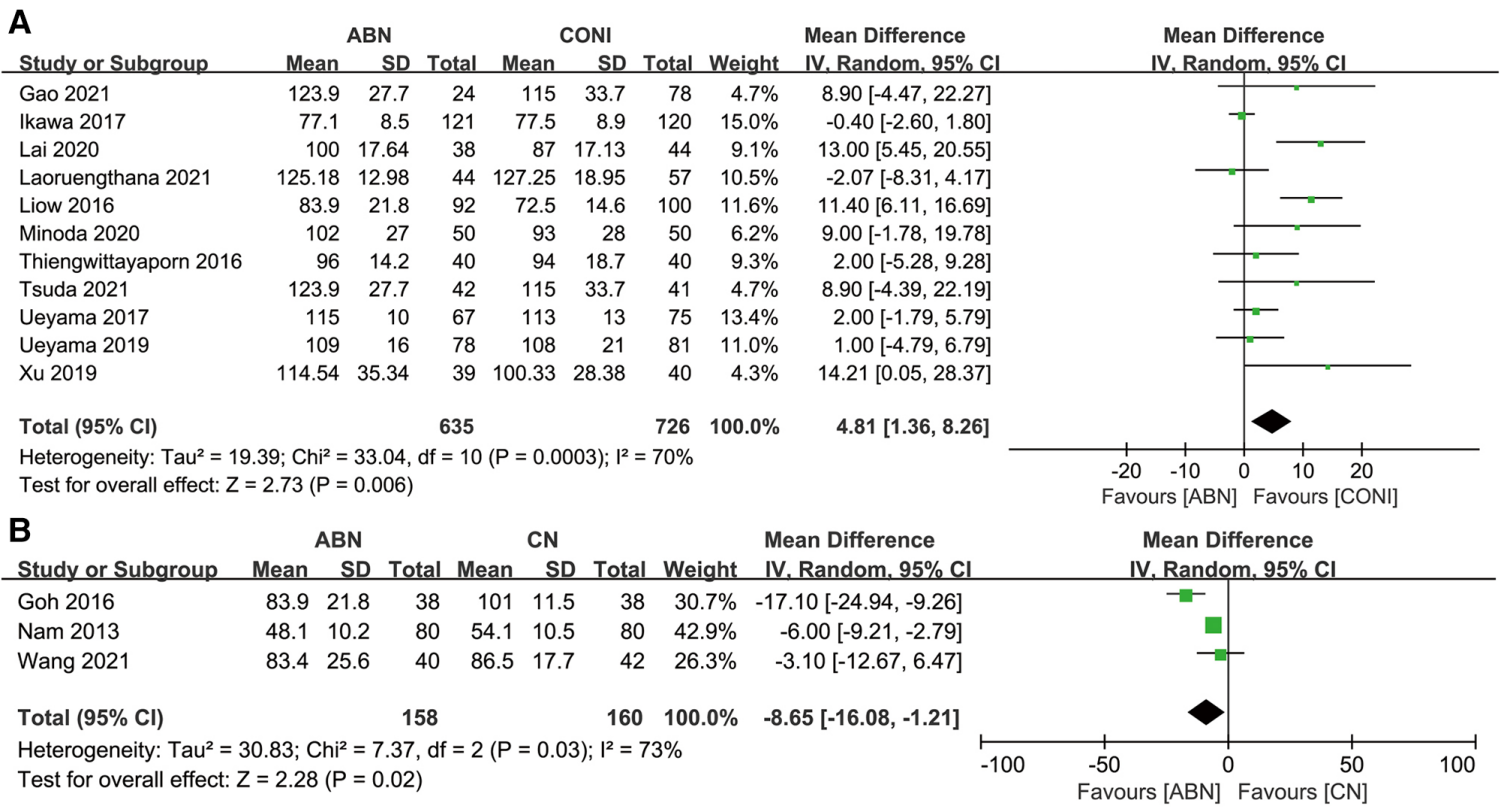
(**A**) Forest plot of surgical duration A: accelerometer-based navigation (ABN) system vs. conventional instrumentation (CONI); (**B**) ABN system vs. computer navigation (CN) system.

For the tertiary outcome on surgical duration, eleven articles assessed the duration required when using the ABN and CONI approach. The results showed that using the ABN system significantly prolonged the surgical time (Figure 12A, MD: 4.81, 95% CI: 1.36 to 8.26, *P* = 0.006, *I*^2^ = 70%). Three studies assessed the required surgical duration when using the ABN and CN systems, and was found that the surgical time with the CN system was significantly longer (Figure 12B, MD: −8.65, 95% CI: −16.08 to −1.21, *P* = 0.02, *I*^2^ = 73%).

The *P* values of all the outcomes from the meta-analysis are shown in Table 3. Statis-tical differences were found between the ABN and CONI groups for HKA, CFA, CTA, SFA, HKA outliers, CFA outliers, CTA outliers, STA outliers, and surgical duration. However, no statistical differences were found between the ABN and CN groups, except for with surgical duration.

**Table 3:** Calculated *P* values for study outcomes.

ABN vs. CONI	HKA	CFA	CTA	SFA	STA	HKA Outliers	CFA Outliers	CTA Outliers	SFA Outliers	STA Outliers	PCO	SD
*P*	<0.0001*	<0.00001*	0.003*	0.007*	0.8	0.00001*	<0.00001*	<0.00001*	0.06	0.0001*	0.34	0.006**
**ABN vs. CN**	**HKA**	**CFA**	**CTA**	**SFA**	**STA**	**HKA Outliers**	**CFA Outliers**	**CTA Outliers**	**SFA Outliers**	**STA Outliers**	**PCO**	**SD**
*P*	0.08	0.36	0.94	NA	NA	0.37	0.11	0.09	NA	NA	0.44	0.02*

ABN, accelerometer-based navigation; CONI, conventional instrumentation; CN, computer navigation; HKA, hip-knee ankle angle; CFA, coronal femoral angle; CTA, coronal tibial angle; SFA, sagittal femoral angle; STA, sagittal tibial angle; PCO, postoperative clinical outcome; SD, surgical duration. *P* < 0.05 represented statistic difference.

* Represented favoring ABN system.

** Represented favoring CONI system.

## Discussion

The main finding of this study is that using an accelerometer-based navigation (ABN) system during total knee arthroplasty improves the accuracy of coronal and sagittal alignments and generates fewer outliers compared with conventional instrumentation. However, the ABN system also prolongs the surgical time and there was no statistical difference in postoperative clinical outcomes. The results also did not show any significant differences in coronal alignments, outliers, and postoperative clinical outcomes between the ABN and CN systems, but using the ABN system resulted in a shorter surgery.

Component alignment is regarded as one of the most crucial factors affecting postoperative functionality and clinical outcomes (4, 5). This study found that using the ABN system resulted in more accurate alignment and fewer outliers than with CONI, which could allow the centers of the femoral head and ankle joint to be more precisely located during surgery. The accelerometer and gyroscope with the ABN system permit the mechanical axis of the lower extremity to be identified, which is then used to guide the resection of the distal femur and proximal tibia on the coronal and sagittal planes. The CONI approach uses an intramedullary guidance system for femoral bone resection, with a rod being used to represent the anatomical axis of the femur. However, the accuracy of the anatomical axis can be affected by the location of the entry point and direction of insertion, as well as femoral deformities such as the sagittal bowing, which can lead to malalignment (43, 44). Also, the alignment accuracy of the femoral and tibial components could be confirmed during the ABN surgery. These characteristics of the ABN system led to more accurate alignment than the CONI approach. However, a longer surgical duration is required when using the ABN system, with 9 of 11 studies showing that the ABN system prolonged the surgical time more than CONI. This may be due to the calculation of the centers of the femoral head and ankle joint which requires the surgeon to swing the leg more than 13 times during the surgery. Moreover, the 3 studies included in the meta-analysis of ABN and CN systems indicated that the CN system required a significantly longer surgical time. A possible reason is the more complicated procedure which required the placements of pin trackers and bone registration. In contrast, the surgical techniques and tools used with the ABN system are more similar to the conventional instrumentation, which most surgeons are familiar with. Compared with conventional instrumentation (CONI), computer navigation (CN) based on image-guidance improves the accuracy of component alignment, but the longer surgical duration than both CONI and ABN might increase the risk of wound complications (45). The additional cost and complications with pin trackers, such as femoral shaft fracture, also limit the widespread application of CN systems (46–48).

The use of ABN and CN systems results in less blood loss for the patient because they do not require intramedullary nailing for navigation (24, 49). This is beneficial for rehabilitation and improving clinical outcomes (49). However, the meta-analysis in this study did not show any statistically significant difference in postoperative outcomes between the ABN system and CONI approach, which is supported by previous studies (18, 19). There is no evidence to indicate that the ABN and CN systems improve long-term clinical outcomes, although they have been shown to improve the accuracy of the alignments. Studies have demonstrated that component rotational alignment plays a key role in knee mechanics and kinematics and can have a demonstratable effect on postoperative clinical outcome (6, 8, 9). Complications like anterior knee pain, patellar subluxation, excessive polyethylene wear, and early failure have been associated with component malrotation. Similarly, errors with internal rotational alignment of the tibial component have been reported as a major cause of knee pain after TKA (7, 50, 51). Unfortunately, none of the studies assessed used an ABN system for rotational alignment on the transverse plane. Future work may consider using an approved ABN system for the rotational alignment of components during TKA surgery, which may further improve clinical outcomes. Kinematical alignment (KA) is a method that differed from the mechanical alignment (MA) for TKA. Previous studies demonstrated that the clinical outcomes of TKA procedures with KA were better than MA (52, 53). However, the requirement for accuracy of bone resection and alignment in KA was much higher. Besides, functional alignment (FA) is a new method based on navigation and robots which aimed to reduce the damage to the soft tissues and enhance rehabilitation, as well as improve clinical outcomes. The ABN may promote the application of KA and FA by achieving a more accurate alignment. An inertial measurement unit (IMU) based on the accelerometer and gyroscope could also be incorporated into a wearable device to record joint activity during postoperative rehabilitation (54).

There are several limitations to this study. First, a small number of studies were included in the meta-analysis, especially when comparing the ABN and CN systems, the differences in sagittal alignments, and outliers between the ABN and CN systems were not analyzed, which might weaken the analysis. While the entire pool of relevant literature that was identified was included in the analysis, the relative novelty of ABN and CN systems meant there were few publications to assess. Besides, the follow-up period of the included studies varied widely, with the longest period being less than 2 years, which could not reliably predict long-term outcomes. Inconsistencies in the observation period may negatively influence the reliability of the pooled results.

## Conclusion

The meta-analysis performed in this study suggested that the ABN system was simi-lar to the CN system in terms of the accuracy of component alignment, but had a longer surgical period. The ABN system also significantly improved the precision of alignments over the CONI approach, although it prolonged the surgical time, whereas the ABN system save the surgical time than the CN system. However, there was no significant difference in the postoperative clinical outcome when using the CONI, ABN, and CN systems.
